# Safety and efficacy of abiraterone acetate in chemotherapy-naive patients with metastatic castration-resistant prostate cancer: an Italian multicenter “real life” study

**DOI:** 10.1186/s12885-017-3755-x

**Published:** 2017-11-10

**Authors:** Luca Cindolo, Clara Natoli, Cosimo De Nunzio, Michele De Tursi, Maurizio Valeriani, Silvana Giacinti, Salvatore Micali, Mino Rizzo, Giampaolo Bianchi, Eugenio Martorana, Marcello Scarcia, Giuseppe Mario Ludovico, Pierluigi Bove, Anastasia Laudisi, Oscar Selvaggio, Giuseppe Carrieri, Maida Bada, Pietro Castellan, Stefano Boccasile, Pasquale Ditonno, Paolo Chiodini, Paolo Verze, Vincenzo Mirone, Luigi Schips

**Affiliations:** 1 Department of Urology, ASL Abruzzo2, Via dei Vestini, Chieti, Italy; 2 Department of Medical, Oral and Biotechnological Sciences, Centro Scienze dell’Invecchiamento e Medicina Traslazionale (CeSI-MeT), Chieti, Italy; 3 Department of Urology, “Sant’Andrea” Hospital , Sapienza University”, Rome, Italy; 4 Radiation therapy Unit, “Sant’Andrea” Hospital, “Sapienza University”, Rome, Italy; 5 Oncology Unit, “Sant’Andrea” Hospital, “Sapienza University”, Rome, Italy; 6 Department of Urology, University of Modena & Reggio Emilia, Baggiovara Hospital, Via Giardini, 1355, Baggiovara, Italy; 7 Ente Ecclesiastico Ospedale “F. Miulli”, S.P. per Santeramo Km 4.100, Acquaviva delle Fonti, Italy; 8 Department Of Experimental Medicine and Surgery, Azienda Policlinico Tor Vergata, Rome, Italy; 9 UOSD of Medical Oncology Azienda Policlinico Tor Vergata, Rome, Italy; 10 Department of Urology, University of Foggia, V.le L. Pinto, Foggia, Italy; 11 Urology and Andrology Unit II, Department of Emergency and Organ Transplantation, University of Bari, Piazza G. Cesare 11, Bari, Italy; 12 Medical Statistics Unit, University of Campania “Luigi Vanvitelli”, via L. Armanni 5, Naples, Italy; 13 Department of Neurosciences, Sciences of Reproduction and Odontostomatology, Urology Unit, University of Naples “Federico II”, Naples, Italy; 14 Department of Urology, ASL Abruzzo2 , “S. Pio da Pietrelcina” Hospital, Via San Camillo de Lellis 1, Vasto, Italy

**Keywords:** Prostate cancer, Androgen deprivation therapy, Abiraterone acetate, Castration-resistant prostate cancer, Androgen receptor

## Abstract

**Background:**

To evaluate the safety and efficacy of abiraterone acetate (AA) in the “real life” clinical practice for men with chemotherapy-naïve metastatic castration-resistant prostate.

**Methods:**

A consecutive series of patients with mCRPC in 9 Italian tertiary centres treated with AA was collected. Demographics, clinical parameters, treatment outcomes and toxicity were recorded. The Brief Pain Inventory scale Q3 was tracked and patient treatment satisfaction was evaluated. Survival curves were estimated by the method of Kaplan-Meier and Cox regression and compared by the log-rank test statistic.

**Results:**

We included 145 patients (mean age 76.5y). All patients were on androgen deprivation therapy. Patients had prior radiotherapy, radical prostatectomy, both treatments or exclusive androgen deprivation therapy in 17%, 33%, 9% and 40%, respectively. 57% of the patients had a Gleason score higher more than 7 at diagnosis. 62% were asymptomatic patients. The median serum total PSA at AA start was 17 ng/mL (range 0,4–2100). The median exposure to AA was 10 months (range 1–35). The proportion of patients achieving a PSA decline ≥50% at 12 weeks was 49%. Distribution of patient satisfaction was 32% “greatly improved”, 38% “improved”, 24% “not changed”, 5.5% “worsened”. Grade 3 and 4 toxicity was recorded in 17/145 patients 11.7% (70% cardiovascular events, 30% critical elevation of AST/ALT levels). At the last follow-up, median progression free and overall survival were 17 and 26.5 months, respectively. Both outcomes significantly correlated with the presence of pain, patient satisfaction, PSA baseline and PSA decline.

**Conclusions:**

The AA is effective and well tolerated in asymptomatic or slightly symptomatic mCRPC in a “real life” setting. The survival outcomes are influenced by the presence of pain, patient satisfaction, baseline PSA and PSA decline.

**Trial registration:**

The study was retrospectively registered at ISRCTN as DOI:10.1186/ISRCTN 52513758 in date April the 30th 2016.

**Electronic supplementary material:**

The online version of this article (10.1186/s12885-017-3755-x) contains supplementary material, which is available to authorized users.

## Background

Prostate cancer (PCa) is the most common male neoplasm and the second leading cause of death from cancer [1].

External beam radiation therapy and surgery are the best options for the treatment of a localized disease, however after an initial treatment with curative intent almost 34% of patients developed progressive metastatic disease [2]. Currently, about 5% of the men were newly diagnosed with metastatic PCa, compared with 20–25% >20 yr. ago [3].

For patients with progressive, recurrent and/or metastatic PCa the androgen deprivation therapy (ADT) is the main therapeutic option, even though the progression to a castration-resistant state invariably occurs after a median time of 18–24 months [3]. The median time from the diagnosis of metastatic disease to death is about 40 months. The metastatic castration-resistant prostate cancer (mCRPC) is the final common pathway in the disease continuum of PCa and remains a clinically relevant phenotype with an elevated burden of mortality. Several mechanisms have been proposed to explain the acquisition of the castration-resistant prostate cancer status including the upregulation of the androgen receptor (AR), induction of AR splice variants, AR point mutations, upregulation of glucocorticoid receptors, activation of alternative oncogenic signaling pathways, neuroendocrine transformation and immune evasion via PD-L1 upregulation [4, 5].

Nowadays several treatments are available for the management of mCRPC prior to chemotherapy. In particular, abiraterone acetate (AA) has been used in several studies and in different clinical settings, demonstrating the reliability and the robustness of the oncological results of AA in terms of overall survival, PSA progression, radiological free survival, time to opiate, etc. [6–12]. Notwithstanding these RCTs, few studies have evaluated the role of AA in managing chemonaive mCRPC in a “real life” setting [12, 13].

The aim of our study was to evaluate the safety and efficacy of AA plus the prednisone regimen in mCRPC chemotherapy-naive patients in an Italian multicentre “real life” study.

## Methods

### Patients and measures

The study was registered at ISRCTN as DOI:10.1186/ISRCTN 52513758. A consecutive series of 145 (November 2013–June 2016) patients with progressive mCRPC and castrate levels of testosterone (<50 ng/dl), chemonaive, treated with AA plus prednisone in 9 Italian tertiary cancer centers were enrolled in a dedicated database (Additional file 1). Patients with visceral metastases were included only if they were not fit for chemotherapy. Four patients, in one center, received AA plus prednisone for compassionate use before the final version of the COU-AA 302 study.

Patients were treated with AA 1000 mg once daily in association with prednisone 5 mg twice a day until progression, death or unacceptable toxicity.

A physical examination, laboratory studies (including a full blood count, routine biochemistry and serum PSA), were carried out at baseline and at visits every 4 weeks. Patients were reviewed every 4 wk. until disease progression occurred or treatment was discontinued for other reasons. Periodic re-evaluation with imaging was performed every 12–16 weeks as required by the Italian Medicine Agency (Agenzia Italiana Farmaco, AIFA) for the AA prescription.

Demographics, clinical parameters, treatment outcomes and toxicity events were recorded. The Gleason score at the diagnosis was recorded. The performance status was measured by the Eastern Cooperative Oncology Group (ECOG) and the pain by the Brief Pain Inventory scale [9]. At the 6 month follow-up visit patients were asked to rate the extent to which they were subjectively improved with the AA treatment on a 4-point, arbitrary, not validated scale. The categories were: 1- greatly improved, 2- improved, 3- not changed, 4- worsened.

Treatment-related toxicity was collected and graded monthly according to the National Cancer Institute Common Terminology Criteria for Adverse Events 4.02 toxicity scale.

Overall Survival (OS) was defined as the time between treatment initiation and either the date of death or of the last follow-up for surviving patients. Progression free survival (PFS) was defined as the time from the first dose of AA to the first clinical (pain, general status) or new radiographic event.

The PSA decline was defined as a response at 12 weeks equal or greater than 50% in the PSA relative to the baseline.

### Statistical analysis

Data were analyzed using SAS 9.2 (SAS Institute Inc., Cary, NC, USA) and R software version 3.1.0 (R Foundation for Statistical Computing, Vienna, Austria). Continuous variables were reported as either mean and standard deviation (SD) or median and range on the basis of their distribution. Comparisons of variables among groups were performed by the one-way ANOVA or Kruskal–Wallis test. Categorical variables were expressed as the absolute number and percentage and analyzed by the Chi-square test. Survival curves were estimated by the product-limit method of Kaplan-Meier and compared using the log-rank statistics. The Cox regression model was used to estimate the hazard ratio (HR) and 95% confidence intervals (CI). An alpha value of 5% was considered as the threshold for significance.

## Results

Overall, 145 patients who initiated AA between November 2013 and June 2016 were enrolled. Table 1 summarizes the characteristics of the patient cohort. In particular, the median age was 76.5 years and 33.8% had already received surgery, whereas 40% of the patients were treated with ADT only. Patients with a Gleason score higher than 7 at diagnosis represented 57.5% of the series. About 38% of patients were symptomatic prior to the initiation of AA, with an ECOG-PS ranging between 0 and 1 in 93% of subjects. Only 11% of patients received more than 2 hormonal manipulations before AA.

**Table 1:** Patient characteristics (*n* = 145)

Variable	Value
Age years, mean (sd)	76.5 (7.0)
ECOG performance status, No. (%) (missing = 14)	
0–1	125 (95)
> 2	6 (5)
Presence of Pain, yes, n (%)	56 (38.6)
Brief Pain Inventory Question #3, >2, *n* (%), (missing =13)	46 (34.9)
Baseline PSA, median (range)	17.4 (0.4 to 2100.0)
Baseline ALT, median (range)	20 (8–87)
Baseline AST, median (range)	18.5 (6–309)
Gleason at time of initial diagnosis, *n* (%) (missing = 4)	
> 7	81 (57.5)
Local treatment, n (%)	
None	58 (40.0)
External Beam Radiation Therapy	25 (17.2)
Radical Prostatectomy	49 (33.8)
Both	13 (8.9)
Disease location, *n* (%) (missing =5)	
Bone only	75 (53.5)
Lymph nodes only	22 (15.7)
Visceral only	4 (2.8)
Prostatic fossa only	11 (7.8)
Multiple sites	28 (20.0)
Comorbidity, *n* (%)	
None	33 (22.7)
Cardiovascular only	50 (35.7)
Metabolic only	8 (5.5)
Multiple (cardiovascular + metabolic)	26 (17.9)
Other	28 (19.3)
Time to mCRPC from initial diagnosis years, median (range)	5.0 (0.2 to 17.7)
Hormonal manipulations before AA >2, *n* (%)	22 (15.2)
Duration of ADT >12 m, *n* (%)	113 (77.9)

Abreviations: *mCRPC* metastatic castration resistant prostate cancer, *ECOG* Eastern Cooperative Oncology Group, *PSA* prostate specific antigen, *AA* abiraterone acetate, *ALT* alanine aminotransferase, *AST* aspartate aminotransferase, *ADT* androgen deprivation therapy

The ADT lasted more than 12 months in 77.9% of patients, with a median time of mCRPC development of 5 years. The median serum total PSA at baseline was 17.4 ng/mL (range 0.4–2100). Overall the median exposure to AA was 10mo (range 1–35) (1 cycle = 1 month), with a 51% rate of dropout (66% for disease progression/clinical deterioration, 14.8% for death, 10.8% lost to follow-up, 8.1% for toxicity) (Table 2). Specifically, relevant toxicity (Grade 3 and 4) was recorded in 17 out of 145 patients (11.7%): 12 had cardiovascular events, 5 had a critical elevation of AST/ALT levels (within the 4th month).

**Table 2:** Treatment details

Variable	Value
N of cycles of AA, median (range)	10 (1–35)
Last PSA, median (range), *n* = 130	9.7 (0.0 to 2743.0)
12 weeks PSA, median (range), *n* = 99	7.7 (0.0 to 900.0)
12 weeks PSA decline, n (%), *n* = 99	49 (49.5)
12 weeks ALT, median (range), *n* = 58	22 (88–215)
12 weeks AST, median (range), *n* = 58	23 (9–150)
Patients’ subjective impression on AA regimen, *n* = 108 (missing =27)	
1 = greatly improved	35 (32.4)
2 = improved	41 (37.9)
3 = not changed	26 (24.0)
4 = worsened	6 (5.5)
Median follow-up time, month (IQR)	13.6 (7–16)
Death, *n* (%)	33 (22.8)
Progression, *n* (%)	56 (38.6)
Median PFS, month (95% CI)	18.5 (16–20)
Median OS, month (95% CI)	26.5 (21–32)

Abreviations: *PSA* prostate specific antigen, *AA* abiraterone acetate, *ALT* alanine aminotransferase, *AST* aspartate aminotransferase, *PFS* progression free survival, *OS* overall survival

**Fig. 1:**
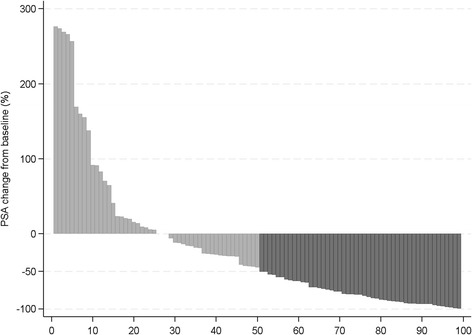
Waterfall plot showing the 12w PSA decline in patients with follow-up >3 months (%). A negative percentage indicates a decline in PSA. A positive percentage indicates that the patient never had a decline in PSA

At the last follow up 50.3% of the patients were still on active treatment with a median PSA of 9,7 ng/mL. Among patients treated for more than 3 months 53.2% achieved a PSA decline ≥50% (Fig. 1).

The patient subjective impression regarding the AA regimen was recorded at a 6 month follow-up visit on 108 patients (27 patients missing) and was described as “greatly improved”, “improved”, “not changed”, “worsened” (32.4%, 37.9%, 24% and 5.5%, respectively) (Table 2). Ten patients abandoned the AA regimen before reaching the 6 month check point.

**Fig. 2:**
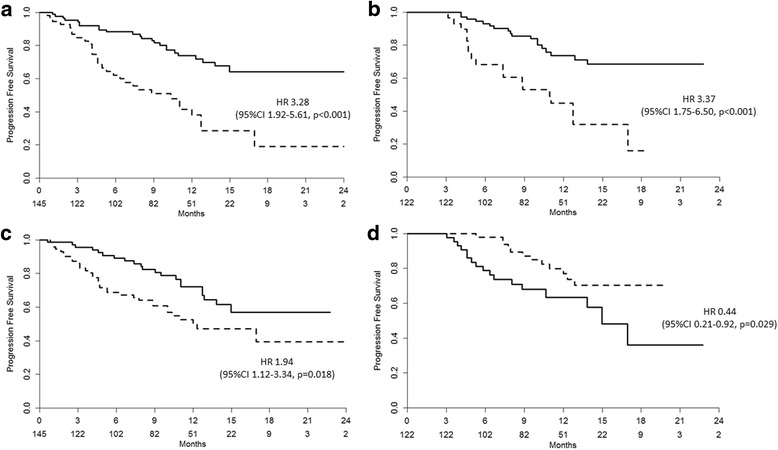
PFS according to different clinical variables: **a**) Pain (solid line = no; dotted line = yes); **b**) patient satisfaction in patients with follow-up >3 months (solid line = satisfied + very satisfied; dotted line = worsened + not modified); **b**) baseline PSA (solid line = <17 ng/ml; dotted line = ≥17 ng/ml); **d**) 12w PSA decline in patients with follow-up >3 months (solid line = <50%; dotted line ≥50%)

The estimated median PFS was 18 months (95% CI 16–20 months). The PFS was significantly associated with patient satisfaction (*p* < 0.001) [HR 3.37 (95% CI 1.75–6.50)], pain (p < 0.001) [HR 3.28 (95% CI 1.92–5.61)], baseline PSA (*p* = 0.018) [HR 1.94 (95% CI 1.12–3.34)] and PSA decline (*p* = 0.029) [HR 0.44 (95% CI 0.21–0.92)] (Fig. 2).

**Fig. 3:**
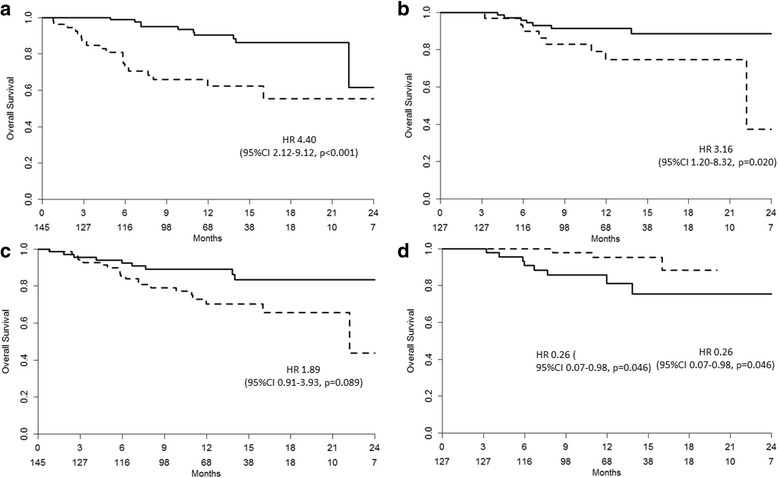
OS according to different clinical variables: **a**) Pain (solid line = no; dotted line = yes); **b**) patient satisfaction in patients with follow-up >3 months (solid line = satisfied + very satisfied; dotted line = worsened + not modified); **c**) baseline PSA (solid line = <17 ng/ml; dotted line = ≥17 ng/ml); **d**) 12w PSA decline in patients with follow-up >3 months (solid line = <50%; dotted line ≥50%)

The estimated overall survival was 26.5 months (95% CI 21–32 months). Overall survival was associated with satisfaction (p = 0.02) [HR 3.16 (95% CI 1.20–8.32)], pain (*p* < 0.001) [HR 4.40 (95% CI 2.12–9.12)] and PSA decline (*p* = 0.046) [HR 0.26 (95% CI 0.07–0.98)] (Fig. 3).

## Discussion

In the current study, we have depicted a representative snapshot regarding the efficacy of AA in an unselected patient population as in a “real life” scenario. Herein, with a mid term follow-up, we confirmed that AA plus prednisone is an effective treatment with excellent patient satisfaction (“greatly improved/improved”: 69.2%) and with a good safety profile (Grade 3 and 4 toxicity recorded in 11.7%). However, in a different setting (real life vs RCT) of different mCRPC patients (older patients, with lower value of baseline PSA, and shorter follow-up) we obtained results in terms of survival outcomes comparable with those reported in the COU-302 trial [6]. In particular, we observed a median OS of 26.5 (95% CI 21–32) versus 34.7 (95% CI 32–36) months in our study. Moreover, our patients received a median of 10 AA cycles (instead of 13.8 in the COU-302 trial) and were followed for 13.6 months (instead of 49.2 in the COU-302 trial), nevertheless the drug related adverse events leading to treatment discontinuation was almost the same (8.1% vs 7%) [6].

Although our trial was not designed to compare the effect of AA vs placebo as in the COU302 trial and comparison with this study is extremely difficult, our experience confirms that AA, in a real life setting, could be safely used to manage patients with chemonaive mCRPC and obtain good results regarding cancer control and patient satisfaction. The phenomenon of variations in terms of efficacy-effectiveness between RCT and real life studies clearly is not specific for AA treatment. In the mCRPC field, similar results have also been reported on the clinical effect of docetaxel in 2013 [14].

In modern oncology a wider space has been recognized as the so-called “Patient Reported Outcomes” (PROs), to warrant that the overall efficacy and safety profiles of new therapies reflect patient experience and perceptions [15, 16]. We think that the patient satisfaction rating scale used in our study, which may be considered a proxy of other and more complex PROs, has given us a new insight into the AA therapy even with its extreme simplicity. Albeit a missing not negligible data rate (27/108), for the first time we analyzed and published the patients’ subjective impression on the AA regimen as a potential predictor of the survival variables documenting a good correlation with both PFS and OS (Figs. 1 and 2) [17, 18].

We also confirmed, as observed in the posthoc analysis of the COU 302 trial [19], that patients with a higher PSA level at baseline and suffering from significant pain at baseline are at a higher risk for an unsatisfactory outcome under AA treatment. Again, herein, even in a real life setting, we demonstrated that some patient characteristics (PSA and pain) better reflect a better response to the treatment; further studies and models are needed to exactly identify which patients mostly benefit from the AA treatment.

Real life data on AA in mCRPC are available in Asian and Danish populations [12, 13]. Unfortunately, our experience is not comparable with the data presented by Poon, considering that they enrolled patients with more advanced and aggressive disease (40% visceral metastatic disease vs 2.8%; and median baseline PSA 212 versus 17 ng/ml). Furthermore, in the Asian study the patients were not followed using a standard protocol and toxicity was retrospectively evaluated without a centralized control, which on the contrary is mandatory in Italy. These different baseline characteristics could explain the main differences observed in terms of dropout rates (39% vs 51%), toxicity requiring AA discontinuation (5.2 vs 11.2%), disease progression rates (64% vs 38.6%) and median PFS (6.7 vs 18.5 months). On the other hand, even the comparison with the Danish population seems to be difficult. We enrolled and treated an older population (76 vs 71 years) with a probably less aggressive disease (baseline PSA 17 vs 156 ng/ml). With a prolonged exposure to the AA (10 vs 5.3 cycles) we recorded a not negligible improvement in overall survival (25 vs 16.6 months) obtaining a better PSA decline control (50% vs 36%). Taken together, these observations suggest that even in a real life setting AA treatment in patients with a less aggressive and less advanced disease in terms of PSA and visceral/nodal metastases is associated with a better outcome, as also highlighted in 2016 by Miller [19] and recently showed by Bögemann [20] during the last ASCO meeting.

We must acknowledge some important limitations to our study. It is a retrospective analysis of a prospective collected database and it includes all the possible limitations of these studies such as the under-reporting of adverse events, incompleteness of data collection and selection biases. However, all these possible drawbacks did not affect the ability to correctly evaluate the survival outcomes, especially due to the peculiar dispensing procedures for AA in Italy. Specifically, the prescription and the dispensation of AA in our country are monthly checked and confirmed in case of clinical benefit without critical toxicity. All these data are collected by physicians and ensure a meticulous observation and report of progressive disease and/or fatal events. The length of the follow-up is another limit of the study and a future report is necessary. Also, the use of a non validated tool to evaluate patient satisfaction regarding treatment should be considered a limitation. When we started our study AA was the only approved drug for mCRPC patients considering that enzalutamide treatment has been available since February 2016. So far we have no real life data on the new available treatment modalities used to manage mCRPC. However, to the best of our knowledge, no studies are available in the literature evaluating enzalutamide, or radium-223 in a real life setting.

Notwithstanding all these limitations, our study represents an early multicentre European real life experience evaluating the effect of AA in mCRPC, and shows that, even in this different clinical scenario, it is associated with a significant effect on oncological and PRO outcomes similar to what has been observed in RCTs, even if further subsequent evaluations were warranted.

## Conclusion

Our data confirm that in a “real life” setting (in a population different in terms of age and comorbidities compared with RCT), AA treatment is effective and safe in mCRPc naïve chemotherapy patients. The survival outcomes are influenced by the presence of pain, patient satisfaction, baseline PSA and PSA decline. A prolonged follow-up is needed to definitely evaluate long term survival outcomes.

## Additional file

Additional file 1: Raw data abiraterone 2017. abiraterone database 2017. clinical data. (DOC 400 kb)

## Abbreviations

95% CI: 95% confidence intervals
AA: Abiraterone acetate
ADT: Androgen deprivation therapy
ALT: Alanine aminotransferase
AR: Androgen receptor
AST: Aspartate aminotransferase
ECOG: Eastern Cooperative Oncology Group,
HR: Hazard ratio
IQR: Interquartile range
mCRPC: Metastatic castration resistant prostate cancer
OS: Overall survival
PCa: Prostate cancer
PFS: Progression free survival
PSA: Prostate-specific antigen

## References

[1] Edwards BK, Noone AM, Mariotto AB, Simard EP, Boscoe FP, Henley SJ (2014). Annual report to the nation on the status of cancer, 1975–2010, featuring prevalence of comorbidity and impact on survival among persons with lung, colorectal, breast, or prostate cancer. Cancer.

[2] Pound CR, Partin AW, Eisenberger MA, Chan DW, Pearson JD, Walsh PC (1999). Natural history of progression after PSA elevation following radical prostatectomy. JAMA.

[3] Pagliarulo V, Bracarda S, Eisenberger MA, Mottet N, Schröder FH, Sternberg CN (2012). Contemporary role of androgen deprivation therapy for prostate cancer. Eur Urol.

[4] Boudadi K, Antonarakis ES (2016). Resistance to novel antiandrogen therapies in metastatic castration-resistant prostate cancer. Clin Med Insights Oncol.

[5] Chandrasekar T, Yang JC, Gao AC, Evans CP (2015). Targeting molecular resistance in castration-resistant prostate cancer. BMC Med.

[6] Ryan CJ, Smith MR, Fizazi K, Saad F, Mulders PF, Sternberg CN (2015). Abiraterone acetate plus prednisone versus placebo plus prednisone in chemotherapy-naive men with metastatic castration-resistant prostate cancer (COU-AA-302): final overall survival analysis of a randomized, double-blind, placebo-controlled phase 3 study. Lancet.

[7] Manokumar T, Aziz S, Breunis H, Rizvi SF, Joshua AM, Tannock IF (2016). A prospective study examining elder-relevant outcomes in older adults with prostate cancer undergoing treatment with chemotherapy or abiraterone. J Geriatr Oncol.

[8] Fröbe A, Murgić J, Rauh S (2016). Single institution long-term efficacy and safety analysis of abiraterone acetate (AA) in the treatment of patients with metastatic castration-resistant prostate cancer (mCRPC) in a named patient programme (NPP). ESMO Open.

[9] Fizazi K, Scher HI, Molina A, Logothetis CJ, Chi KN, Jones RJ (2012). Abiraterone acetate for treatment of metastatic castration resistant prostate cancer: final overall survival analysis of the COU-AA-301 randomised, double-blind, placebo-controlled phase 3 study. Lancet Oncol.

[10] Satoh T, Uemura H, Tanabe K, Nishiyama T, Terai A, Yokomizo A (2014). A phase 2 study of abiraterone acetate in Japanese men with metastatic castration-resistant prostate cancer who had received docetaxel-based chemotherapy. Jpn J Clin Oncol.

[11] Taplin ME, Montgomery B, Logothetis CJ, Bubley GJ, Richie JP, Dalkin BL (2014). Intense androgen-deprivation therapy with abiraterone acetate plus leuprolide acetate in patients with localized high-risk prostate cancer: results of a randomized phase II neoadjuvant study. J Clin Oncol.

[12] Poon DM, Chan K, Lee SH, Chan TW, Sze H, Lee EK (2016). Abiraterone acetate in metastatic castration-resistant prostate cancer - the unanticipated real-world clinical experience. BMC Urol.

[13] Thortzen A, Thim S, Røder A, Brasso K (2016). A single-centre experience with abiraterone as treatment for metastatic castration-resistant prostate cancer. Urol Oncol.

[14] Templeton AJ, Vera-Badillo FE, Wang L, Attalla M, De GP, Leibowitz-Amit R (2013). Translating clinical trials to clinical practice: outcomes of men with metastatic castration resistant prostate cancer treated with docetaxel and prednisone in and out of clinical trials. Ann Oncol.

[15] Scher HI, Halabi S, Tannock I, Morris M, Sternberg CN, Carducci MA (2008). Prostate cancer clinical trials working group. Design and end points of clinical trials for patients with progressive prostate cancer and castrate levels of testosterone: recommendations of the prostate cancer clinical trials working group. J Clin Oncol.

[16] Gnanasakthy A, Lewis S, Clark M, Mordin M, DeMuro C (2013). Potential of patient-reported outcomes as nonprimary endpoints in clinical trials. Health Qual Life Outcomes.

[17] Cindolo L, Natoli C, De Nunzio C, De Tursi M, Valeriani M, Giacinti S, Micali S, Rizzo M (2017). Abiraterone acetate for treatment of metastatic castration-resistant prostate cancer in chemotherapy-naive patients: an italian multicenter ‘real-life’ 1-year study. Anticancer Res.

[18] Cindolo L, Natoli C, De Nunzio C, De Tursi M, Valeriani M, Giacinti S, Micali S, Rizzo M (2017). Abiraterone acetate for treatment of metastatic castration-resistant prostate cancer in chemotherapy-naive patients: an Italian analysis of Patients' satisfaction. Clin Genitourin Cancer.

[19] Miller K, Carles J, Gschwend JE, Van Poppel H, Diels J, Brookman-May SD (2016). The phase 3 COU-AA-302 study of abiraterone acetate (AA) in men with chemotherapy (CT)-naïve metastatic castration-resistant prostate cancer (mCRPC): stratified analysis based on pain, prostate-specific antigen (PSA) and Gleason score (GS). Eur Urol Suppl.

[20] Bögemann M, Hatzinger M, Hercher D, Matus G, Evaraert EG, Dopchie C, Sheenan D, et al. Real-world treatment with abiraterone acetate in patients with chemotherapy-naïve metastatic castration-resistant prostate cancer (mCRPC). J Clin Oncol. 2017; 35: suppl 6S; abstract 239.

